# Clarifying Hackathon in Health Profession Education

**DOI:** 10.12669/pjms.40.7.10104

**Published:** 2024-08

**Authors:** Azadeh Rooholamini, Junaid Sarfraz Khan, Parvin Rezaei-Gazki, Mahla Salajegheh

**Affiliations:** 1 Dr. Azadeh Rooholamini Department of Medical Education, Medical Education Development Center, Kerman University of Medical Sciences, Kerman, Iran; 2 Prof. Dr. Junaid Sarfraz Khan Dean/CEO, Lady Reading Hospital Medical Teaching Institution, Peshawar, Pakistan; 3 Dr. Parvin Rezaei-Gazki Department of Medical Education, Medical Education Development Center, Kerman University of Medical Sciences, Kerman, Iran; 4 Dr. Mahla Salajegheh Department of Medical Education, Medical Education Development Center, Kerman University of Medical Sciences, Kerman, Iran

Hackathons are a type of opening innovation (OI) that has rapidly expanded globally since 2000. At the beginning of the 21st century, with a paradigm shift towards OI, hackathons as emerging innovation events have changed the way innovation processes within and outside educational institutional boundaries and becoming more and more popular in practice. Today, they are more widely used in business process management, software engineering, and computer science education more like a tool to innovate new solutions.

The term hackathon is a combination of the words “hack” and “marathon”, where individuals with different professional backgrounds work in teams within a set timeframe, usually ranging from a few hours to a few days, to complete projects with creative solutions or prototypes. In other words, “hackathon,” refers to an event where participants come together to ideate, cooperate, and present a solution to a problem or develop innovative projects. The participants in the hackathon can be a diverse range of individuals according to the purpose and topic of the hackathon and having experience and knowledge in that field.^1^

Hackathons can take place in physical locations, such as conference centers or university campuses, or be held virtually, allowing participants from different locations to join remotely. The event structure requires participants to engage in team formation, problem identification, and preparation for the presentation and demonstration of their solution which aligns with the Double Diamond design process. Based on the Double Diamond design process, the hackathon participants follow typical divergence–convergence patterns:

- Discover, that focus lies on divergent activity with forming a team and finding a problem,
- Define, which includes an understanding of the problem and converge into a problem formulation to define the aims and requirements of the design and constraints on the solution,
- Develop, which describes another divergent set of activities to ideate and embody solutions,

Deliver, in which teams focus on converging their solution ideas into a final artifact. Deliver is closely linked with the Develop phase, and often observed rotation between the two phases to synthesize the final design in the design process throughout the hackathon.^1^

Hackathons provide a solution-oriented environment for participants to collaborate, learn from others, and network with like-minded individuals. These events offer opportunities for teamwork, creativity, critical thinking, and fostering a sense of innovation among participants. Participants help each other across the team boundaries free from hierarchical constraints. A competitive type of event, on the other hand, may not show this kind of behavior.^2^

The incorporation of hackathon in health profession education enables academic contexts to utilize them for their innovation as the result of collaborative processes in interdisciplinary teams. Educational hackathons can enhance problem-solving, interest, and creativity in learners as they engage them in the learning process and retention of new knowledge. Also aid learners to promote innovative thinking, improve technical and soft skills, and successful networking.^3^

These events are a constructivist learning model, peer-learning, and collaborative learning experience that can be effective in increasing learner motivation, confidence, and learning. The hacking events give participants an opportunity for experiential learning through learning by doing. They work together to solve problems to maximize their creative abilities and active engagement. Therefore, student engagement was one of the other desired outcomes of the hackathons. One possible reason for this may be that learners found it easy to interact with themselves without the barriers of the teacher-centered approach to knowledge construction.^3^

In an educational context, both educators and learners are motivated to deal with social and environmental issues outside their academic pursuits. Learners teach themselves how to overcome difficulties, learn new skills, and have community involvement during hackathons, and educators as hackers encourage and facilitate the use of systematic design processes on how to design a hackathon.^1^

Hence, developing and introducing hackathon-based pedagogies can be utilized in the design of the curriculum to support traditional classrooms as an extracurricular activity. This curriculum which includes hackathons provides a dynamic and immersive learning experience, encouraging participants to think creatively, collaborate effectively, and develop practical skills.
